# The role and relevance of asymptomatic healthcare worker testing in COVID-19 hospital outbreaks

**DOI:** 10.1017/ash.2022.128

**Published:** 2022-05-16

**Authors:** Matthew Garrod, Katy Short, James Zlosnik, Natalie Prystajecky

## Abstract

**Background:** Many healthcare facilities have faced the decision of conducting point prevalence testing (PPT) of healthcare workers (HCW) during COVID-19 outbreaks. As a containment strategy, PPT can identify asymptomatic or presymptomatic cases for isolation. It is less clear how useful testing asymptomatic HCW is in understanding the spread and possible routes of transmission in an outbreak. This study investigated HCW cases identified through PPT during acute-care outbreaks in Fraser Health (FH), British Columbia, incorporating both epidemiological and whole-genome sequencing (WGS) data to determine their epidemiological source. **Methods:** This study was a retrospective review of cases associated with COVID-19 acute-care outbreaks in FH occurring between December 2020 and June 2021, when most of these infections were of the alpha and gamma lineages. All patients and HCWs with a positive COVID-19 test and epidemiologically linked to the outbreaks were included in the study. WGS results supported determination of epidemiological source for cases. The proportion of patient and HCW cases related to the outbreak was compared. All analyses were conducted using SAS version 4.3 software with the PROC GLM package. **Results:** Between December 2020 and June 2021, 49 acute-care COVID-19 outbreaks were declared. Point-prevalence testing of HCWs, in addition to routine patient PPT, was conducted in 28 outbreaks (57%), with 2,167 eligible HCWs (63%) tested. Testing identified 14 previously unknown HCW cases, representing 12.96% of all HCW cases epidemiologically linked to the outbreaks. None of these HCWs were determined to be the index case for their associated outbreak. There was no statistically significant difference between HCWs and patients regarding WGS failure rate, and all failed samples were removed from further analysis. Patients were 3.8 times as likely as HCWs to present as symptomatic when testing positive. HCWs were 2.2 times as likely as patients to have WGS results unrelated to the outbreak. **Discussion:** Although point-prevalence testing of HCW identified previously unknown cases, these cases were more likely than patients to be unrelated to the outbreak and therefore less useful in understanding the epidemiology of the outbreak. It is difficult to determine whether HCW PPT was effective at preventing transmission, especially with robust infection prevention measures already in place. Patients were more likely than HCWs to present as asymptomatic, however this may be due to the attribution of symptoms to other conditions. **Conclusions:** Point prevalence testing of HCWs during COVID-19 outbreaks may assist with preventing transmission but is less likely to contribute meaningful information to the investigation.

**Funding:** None

**Disclosures:** None

